# The Impact of Health Insurance on Healthcare Utilization by Migrant Workers in China

**DOI:** 10.3390/ijerph17061852

**Published:** 2020-03-12

**Authors:** Fei Zhang, Xinjie Shi, Yun Zhou

**Affiliations:** 1 College of Economics and Management, Zhejiang Normal University, Jinhua 321004, China, zhangfeizju@126.com; 2 China Academy for Rural Development (CARD), School of Public Affairs, Zhejiang University, Hangzhou 310058, China; zysimone@zju.edu.cn

**Keywords:** medical insurance, healthcare service, migrant workers, China

## Abstract

Health insurance is an essential instrument to ensure equal access to medical resources and promote the health of the general population. Robust evidence regarding whether migrant workers have benefited from available insurance schemes is limited. Drawing on survey data from the Rural Urban Migration in China (RUMiC) Project, this paper examines the effects of health insurance on migrant workers’ utilization of routine medical services, the medical burden, and the utilization of preventive medical services using a two-part model, the Heckman model, the Tobit model, and a probit model. Our findings indicate that, first, participating in medical insurance increases migrant workers’ probability of visiting a doctor. Unlike other medical insurance programs that positively affect migrant workers’ medical expenditure, the new rural cooperative medical system fails to play an effective role. Second, participation in any medical insurance program effectively reduces migrant workers’ medical burden and can improve the probability of preventive medical service utilization. Third, self-reported health and disease severity are pivotal to determining migrant workers’ medical expenditure. Fourth, high-income people have a good health status and a lower probability of becoming ill and can afford relatively higher medical expenses once they become ill. China’s medical insurance appears to mainly serve to reduce the financial burden for serious illnesses, reflecting important policy implications for policy-makers.

## 1. Introduction

Since the 1980s, China has gradually established a new basic social health insurance (SHI) system in rural and urban areas, with the major types of insurance including the New Rural Cooperative Medical Scheme (NRCMS), which insures the large rural population; Urban Resident Basic Medical Insurance (URBMI), which targets urban workers in the informal sector and unemployed urban residents; and Urban Employee Basic Medical Insurance (UEBMI), which covers employees in the formal urban sector [1]. In 2009, the central government of China announced a comprehensive reform of the health system, with the ultimate goals of achieving universal coverage for basic healthcare and “establishing a basic healthcare system covering all the population by 2020” [2]. Since then, remarkable progress in health system reform has been achieved. By the end of 2011, more than 90% of the Chinese population was covered by the three main health insurance schemes described above, and the use of healthcare in general has increased significantly [3]. Furthermore, the proportion of out-of-pocket (OOP) payments among total medical expenditure decreased from 60% in 2000 to 40% and 28% in 2008 and 2016, respectively [4].

The migration of rural laborers into cities for employment has been one of the main driving forces of China’s economic growth over the past three decades [5]. These migrants’ health status is a key determinant of their earnings, especially for those engaged in labor-intensive jobs, since maintaining good physical health leads to a stable job, more work hours, and a better salary. However, due to their self-capacity and social policies, migrant workers tend to be stuck in the secondary labor market where they work and live in low-income environments, receive low earnings, engage in dirty and dangerous work, and are vulnerable to injuries and disease [6]. Good health and universal health coverage can be understood as a basic human right and a basic function of the government. By reducing the economic threshold for medical treatment and increasing the availability of medical services, medical insurance has a positive effect on national health and longevity.

Medical and resident health issues have been some of the most concerning problems for the government, scholars, and the general public in recent years. After 2003, with the establishment of a new national medical insurance system, most rural-to-urban migrant workers became qualified for one or more insurance schemes, such as the UEBMI, URBMI, NRCMS, commercial insurance, and/or other local insurance programs. However, robust evidence on whether migrant workers have benefited from these insurance schemes is limited. Meanwhile, the demographic dividend builds on health, reflecting the continuous advancement of China’s industrialization and urbanization [7]. Therefore, this paper studies the relationship between the health of migrant workers and SHI utilization.

The rest of this paper is organized as follows. Section 2 provides a thorough literature review in the field of effects on SHI. Section 3 depicts the model specifications. Section 4 describes the data sources and provides the descriptive statistics for the key variables considered in our econometric estimation. Section 5 discusses the empirical results. The final section concludes the paper and provides recommendations for policy implementation in China.

## 2. Literature Review

Numerous studies have evaluated the effect of medical insurance implementation from two aspects: the medical service utilization effect and the health effect. Most studies have concluded that medical insurance can promote the utilization of medical services [8,9]. However, the findings on the health effect are controversial. For instance, by reviewing the literature on medical insurance, medical service utilization, and health from the past 25 years, Hadley (2003) found that, in some studies on mortality, having medical insurance can reduce the mortality rate by at least 4‒5% [10]. In contrast, Finkelstein and McKnight (2008) found that the Medicare in the United States failed to significantly reduce the overall elderly mortality in its first 10 years [11].

Additionally, many studies have evaluated the implementation effects of UEBMI, URBMI, the NRCMS, commercial insurance, and other local insurance programs in China from different perspectives [12,13,14,15]. For example, Yang and Wu (2014) confirmed that the NRCMS has no effect on reducing OOP outpatient expenses [16]. Regarding the impact of China’s medical insurance on the utilization of medical services among migrant workers, only few comprehensive studies have been conducted. Zhou et al. (2013) found that UEBMI and URBMI but not the NRCMS improved accessibility to preventive medical services for migrant workers, but UEBMI, URBMI, and the NRCMS enhanced routine medical service utilization [17]. Furthermore, Qin et al. (2014) confirmed that UEBMI played a prominent role in reducing the OOP ratio for migrant workers, thus increasing the number of physical examinations and improving their health status; NRCMS has significantly improved the health awareness of migrant workers and their utilization of preventive medical services [1].

The existing literature has not given sufficient attention to the utilization of SHI and medical services among migrant workers. Although the literature indicates that migrant workers are vulnerable in the current reform of the medical security system, little empirical evidence is available on migrant workers’ participation in SHI and the effect of SHI on medical service utilization among migrant workers because collecting representative nationwide data on migrant workers is difficult. In addition, the construction of a medical insurance system for migrant workers remains under exploration.

Based on above foundation, this paper studies the impact of medical insurance on migrant workers’ annual medical expenditure, medical burden, and utilization of preventive medical services. Moreover, we believe that, compared with health effects, the effect of using medical services has more policy reference value for the following three reasons: (1) Promoting health is the ultimate goal of the medical and health system, but the factors affecting health extend beyond the system. Individual health is determined by a series of observable and unobservable individual characteristics and environmental factors, such as genetics, family income, and individual health behaviors. (2) The impact of SHI on health is indirect and uncertain, mainly reducing social risks rather than improving health, while the starting point of SHI is mainly to “improve the entire population’s health status.” (3) The health effect is related to the health indicators evaluated.

Therefore, this paper focuses on the effect of using medical services. Specifically, we propose the following relationships: (1) if SHI has a positive effect on medical expenditure, then SHI promotes migrant workers’ utilization of routine medical services; (2) if SHI has a negative effect on the proportion of annual OOP medical expenditure, then SHI reduces the medical burden of migrant workers; and (3) if SHI has a positive effect on preventive medical services, then SHI promotes migrant workers’ utilization of preventive medical services.

## 3. Methods

In reality, two cases involve zero medical expenditure: in one case, an individual is in good health and does not need medical services, and in the other case, due to the price of medical services or accessibility to medical services, medical expenses should be incurred but are not; that is, no medical expenses are incurred as a result of self-selection. Accordingly, referring to existing research, a two-part model (TPM) and the Heckman selection model are used to obtain unbiased estimates. The impact of medical insurance on the medical burden of migrant workers and their use of preventive medical services is based on the Tobit model and a probit model, respectively.

### 3.1. TPM

A TPM divides the generation of individual medical expenditure into two subprocesses: illness and attendance. The model assumes that the decision of whether to use medical services is a sequential and independent process. Specifically, the first part of the model predicts the probability of illness, which is specified as a probit model: $P\left(P_{i}=1\right)=P(\alpha_{1}Ins_{i}+\beta_{1}x_{i}^{\prime }+\varepsilon_{1i}>0)$, where *P_i_* is a dummy variable equal to 1 if individuals are associated with nonzero medical expenditure and 0 otherwise. $I{ns}_{i}$ represents the status of participating health insurance, and $x_{i}$ is a vector of individual characteristics such as age, sex, education, and income. We assume that $\varepsilon_{1i}∼N\left(0,\sigma^{2}\right)$, and we use a maximum likelihood method for the estimation.

The second part of the model is: $E[log(y_{i}|P_{i}=1)]=\alpha_{2}Ins_{i}+\beta_{2}Z_{i}+\varepsilon_{2i}E[log(y_{i}|P_{i}=1)]=\alpha_{2}INS_{i}+\beta_{2}Z_{i}+\varepsilon_{2i}$ where $y_{i}$ denotes medical expenses and *Z_i_* is a vector of other independent variables besides medical insurance, $\varepsilon_{2i}∼N\left(0,\sigma^{2}\right)\varepsilon_{2i}∼N\left(0,\sigma^{2}\right)$.

The advantage of the TPM model is that it does not require the same set of covariates in both parts. Since some variables that affect the probability of visiting a doctor do not necessarily have an impact on medical expenditure, the two parts of the TPM model in this paper have different variables.

### 3.2. Heckman Selection Model

The sample selection model assumes that all the samples should have medical expenses, and zero medical expenses are the result of the sick samples choosing not to seek medical assistance. Such self-selection behavior will cause sample selection deviation. We use Heckman’s (1979) two-stage method to resolve the problem [18].

At the first stage, the number of migrant workers choosing to receive treatment is taken as a binary dependent variable in the decision-making equation. The probability of migrant workers choosing to seek treatment can be constructed as a binary choice, shown as:

(1) $$P_{i}^{*}=\gamma_{1}Ins_{i}+\lambda_{1}x_{i}^{\prime }+\mu_{1i}>0,\;P_{i}=1;\;\mathrm{otherwise},\;P_{i}=0\;P_{i}=1,log\;y_{i}=\gamma_{2}Ins_{i}+\lambda_{2}z_{i}^{\prime }+\mu_{2i}\;\mu_{1i},\mu_{2i}∼N\left(0,\sigma^{2}\right).P_{i}=1\;P_{i}=0$$

### 3.3. Tobit Model

Since the value of medical burden ranges from 0 to 1, OLS can result in estimation bias. Therefore, we employed the Tobit model to estimate the function: $y_{i}^{*}=\beta^{T}x_{i}+e_{i},e_{i}∼N\left(0,\sigma^{2}\right),i=1,2,\ldots ,n$, $y_{i}=y_{i}^{*}$ if $y_{i}^{*}>0$, $y_{i}=0,$ if $y_{i}^{*}\leq 0$,where $y_{i}^{*}$ is the latent dependent variable, $x_{i}$ is a vector of independent variables that affect migrant workers’ medical burden, and $y_{i}$ $y_{i}$ is the migrant workers’ financial burden of treatment.

### 3.4. Probit Model

Similar to the utilization of formal medical services, the demand for preventive medical services is also caused by the demand for health. However, the obvious difference between preventive medical services and formal medical services is that the consumption of preventive medical services does not improve individual health immediately because of a lag effect. Because the occurrence of disease is random, the consumption of preventive medical services can reduce the incidence of disease and have an impact on the marginal yield rate of formal medical services, and the importance of preventive healthcare has received increasing attention.

The binary probit model for migrant workers’ utilization of preventive medical services is:

(2) $$P_{i}^{*}=\alpha Ins_{i}+\beta z_{i}^{\prime }+\mu_{i},\;\mathrm{if}\;\alpha Ins_{i}+\beta Z_{i}^{\prime }+\mu_{i}>0,\;\mathrm{then}\;P_{i}=1,$$

where $P_{i}^{*}$ is the probability of migrant workers seeking preventive medical services, $Ins_{i}$ represents the status of participating in health insurance, and $Z_{i}$ is a vector of other independent variables.

## 4. Data

### 4.1. Data Source

We used survey data from the Rural Urban Migration in China (RUMiC) Project to comprehensively examine the impact of health insurance participation on the migrant worker population. This project is a national survey designed by a team of international researchers from China and Australia. This project aims to investigate the income, social welfare, quality of life, and other living conditions of rural‒urban migrants [19]. The RUMiC Project was conducted in 2008 and 2009 in 15 different cities with a relatively high concentration of migrants distributed in nine provinces and municipalities of China. Presently, the RUMiC database is considered one of the best national survey databases on rural‒urban migration in China [20]. More information on the design, sampling procedures, and methodology of this project has been documented elsewhere [21,22]. For the purposes of the study, migrant workers between 16 and 65 years old were selected as the research objects. After the elimination of missing values and extreme outliers, 7300 valid samples were obtained.

Of the total sample, 35.15% (sample size: 2566) did not incur any medical expenses in the year; for the 4734 migrant workers who incurred medical expenses, 150 were completely covered for medical expenses, 4188 covered medical expenditure completely at their own expense, and another 546 reported different levels of medical subsidies; that is, 7.48% of the total sample received medical subsidies. The annual average medical expenditure of the entire sample was 574.08 yuan, and the annual average medical expenditure of the sick sample was 885.25 yuan. Of the total sample, 15.04% had experienced illness in the past three months, 13.75% had incurred medical expenses in the past three months, and 25.55% had received a physical examination or been immunized. For those who were sick in the past three months, 45.76% chose to take medicine, 13.13% went to an infirmary or small clinic to see a doctor, and 18.23% went to a hospital to see a doctor. The proportion of seriously ill migrant workers (sample size: 306) who chose to go to the hospital was only 38.24%.

### 4.2. Variable Specification

The dependent variables selected for our analysis were (1) formal medical utilization, where “total medical expenditure in the previous year” was used for measurement; (2) migrant workers’ financial burden of treatment, where the ratio of OOP payments among total medical expenditure in the previous year was used for measurement; and (3) preventive medical service utilization, which was measured by “whether a migrant worker had any physical exams in the previous year.”

Regarding the variable of interest—health insurance—two situations are described in the literature: one is the setting of a binary variable of whether a migrant worker participates in any health insurance program, and the other is the setting of multiple classified variables, that is, the specific health insurance program in which migrant workers participate. We follow the second approach because participation policies, reimbursement mechanisms and the financing levels of various types of health insurance are different. If we simply treat health insurance as a binary variable, the difference between the effects of different types of health insurance will be hidden. Therefore, according to the condition of sample migrant workers’ participation in health insurance, a categorical variable called “type of medical insurance” was constructed, and responses ranged from 0 to 5, representing “no insurance,” “insured by NRCMS,” “insured by employment medical care,” “insured by public health services” (“paid for by the government”), “insured by other single medical insurance” (insured by one program that has not been mentioned above),, and “multiple insurance” (“insured by more than one medical insurance program”), respectively.

Aside from the indicator of health insurance status, we also considered a set of factors that may influence the dependent variables, as summarized in Table 1. These factors included age, sex, education, family income, health status, and morbidity, as commonly used in the literature. We used self-rated health (SRH) to present the health status of migrant workers, which is classified into five levels [23], and other studies show that such a subjective health rating is a good indicator of a respondent’s actual health status. Regarding morbidity, migrant workers were asked, “Were you sick or injured in the last three months? (including chronic or acute disease)”; according to their responses, morbidity was categorized into three levels: 1 represented slightly or not sick, 2 represented fairly sick, and 3 represented seriously sick. Hukou is one of the institutional barriers in the current insurance platforms that affects migrant workers’ participation and use of insurance [1]. According to the RUMiC Project, hukou was categorized into two types: 1 represented local rural household registration, and 2 represented migrant rural household registration. Long working hours had significant adverse effects on most health outcomes, and long working hours were associated with a depressive state, anxiety, sleep conditions, and coronary heart disease [24]. Therefore, these variables were also considered.

### 4.3. Descriptive Statistics

Table 1 reports the descriptive statistics for the sample. A total of 65.95% of the migrant workers were covered by a single insurance program, 3.81% were covered by two or more types of insurance, and 30.25% did not have any medical insurance. When asked about the reasons for not having medical insurance, 35% of the uninsured workers believed that insurance is not necessary to purchase, 27% did not know the details of medical insurance, and 16% could not afford the expense.

Among the sample participants in a single medical insurance program, the NRCMS was the most common insurance held (55.27%), while the other types of medical insurance with a high participation rate included employment medical care (4.62%) and public health services (2.22%). The total number of migrant workers participating in the NRCMS accounted for 58.58% of the sample (including people with multiple insurance programs). The main reason for the high proportion of participation in the NRCMS is that opinions on the establishment of the new rural cooperative medical care system clearly stipulate that the NRCMS must regard a family as a unit of participation. The implementation policy assumes that “a village can leak households, but households do not leak people.” Although many migrant workers leave the countryside to work in cities, they still participate in the NRCMS, which may ensure that other family members can benefit from the NRCMS.

## 5. Empirical Results

Table 2, Table 3, Table 4 and Table 5 report the main estimation results. Table 2 reports the TPM regression results for migrant workers’ medical expenditure. Table 3 reports the regression results of the Heckman selection model. Table 4 reports the Tobit regression results for migrant workers’ medical burden. Table 5 reports probit regression results for migrant workers’ utilization of preventive medical services.

### 5.1. TPM Regression Results for Migrant Workers’ Medical Expenditure

Table 2 reports the TPM regression results for migrant workers’ medical expenditure. We first focus on the impact of various types of medical insurance. After controlling for other variables, employment medical care, the NRCMS, and repeated participation in insurance had significantly positive effects on increasing the probability of migrant workers’ seeking medical consultations. While these items had significantly different effects on migrant workers’ medical expenditure, the coefficient of the NRCMS was negative and statistically significant; in contrast, the coefficients of employment medical care, other medical insurance, and repeated participation in medical insurance were positive and statistically significant, especially for repeated participation in medical insurance. The possible reasons are that the NRCMS requires migrant workers to cover their medical expenses and receive reimbursement after returning to their hometowns, which is a cumbersome reimbursement procedure for migrant workers, and migrant workers have little knowledge about the NRCMS, such as the reimbursement ratio, reimbursement procedures, and reimbursement restrictions. Compared with the NRCMS, other insurance programs have no reimbursement procedures in different places. Additionally, repeated participation in medical insurance expands the reimbursement range. Therefore, participation in insurance—except for the NRCMS—significantly improves migrant workers’ utilization of routine medical services.

The impacts of other control variables on the probability of migrant workers receiving medical consultations and incurring medical expenses are as follows:

(1) SRH and disease severity. Table 2 suggests that, if the SRH of migrant workers is better, then the probability of migrant workers receiving medical consultations and incurring medical expenses is lower after controlling for other variables. The probability of receiving medical consultations and incurring medical expenses increases among migrant workers with severe diseases compared with those who were “healthy or less ill.”

(2) Sex. Contrary to Lü and Wang (2012) [25], we found that women had higher probabilities of receiving medical consultations and incurring medical expenses than men. Female migrant workers face more health risks than male migrant workers; therefore, household medical resource allocation is biased toward women.

(3) Age. The impacts of age on the probabilities of migrant workers receiving medical consultations and incurring medical expenses are positive (the former is not statistically significant), indicating that the depreciation of health capital increases with age. Elderly individuals must spend more on medical expenses to compensate for health capital depreciation to maintain their health.

(4) Education. With increasing education, the probabilities of migrant workers receiving medical consultations and incurring medical expenses increase (the latter is not statistically significant). On the one hand, highly educated residents tend to have higher incomes and can pay for medical services; on the other hand, they usually have more medical information and can efficiently improve their health [26].

(5) Family income. In the overall sample, household income has a statistically significant negative effect on the probability of migrant workers’ receiving medical consultations, while the impact on medical expenditure is statistically significantly positive, indicating that household income has a twofold effect. The health effect suggests that high-income people may have a good health status and a lower probability of becoming ill, and the wealth effect indicates that they have the ability to pay for medical expenses once they become ill, which is consistent with Liu et al. (2003) [27]. These findings also suggest that low-income people face budget constraints in medical consumption, leading them to forgo medical treatment after becoming sick.

### 5.2. Heckman Regression Results for Migrant Workers’ Medical Expenditure

The inverse Mills ratio (IMR) of the Heckman model was statistically significant at the 1% significance level, indicating that the samples exhibit self-selection; that is, some migrant workers should seek medical treatment but do not.

The results of the Heckman model shown in Table 3 are similar to those of the TPM model shown in Table 2. While the coefficients of the NRCMS, family income, and regional characteristic variables decreased and were statistically nonsignificant, the coefficients of other variables increased and were statistically significant, and working hours showed no crowding out effect on medical treatment.

### 5.3. Tobit Regression Results for Migrant Workers’ Medical Burden

Compared to migrant workers who do not participate in any medical insurance, those participating in employment medical care, public health services, the NRCMS, and other medical insurance and those with repeated participation in insurance have a significantly lower medical burden, as shown in Table 4.

Table 4 also shows that migrants with good SRH and a high household income have low medical burdens. However, the coefficients of age and disease severity seem counterintuitive, indicating that the proportion of OOP medical care costs decreased with age and disease severity. As shown by the TPM and Heckman results, medical expenditure increased with disease severity. For SHI, a reimbursement line exists, which dictates that people can receive reimbursement only when medical expenses for critical illness and inpatient services reach a certain reimbursement threshold. Therefore, for mild illnesses, people need more outpatient services, pay low outpatient OOP costs, and obtain low reimbursements or even no medical insurance subsidies, but for serious illnesses, medical expenses increase up to the reimbursement line such that people can receive medical subsidies. In addition, elderly people have a higher probability of critical illness than their younger counterparts. For these reasons, the proportion of medical reimbursement increases with age and disease severity.

Furthermore, we found that among migrant workers who have been reimbursed by SHI, 47 people in a “severely ill” condition spent 6308.02 yuan on annual medical expenditure and 3372.34 yuan on annual OOP costs on average and that these values were far higher than the average annual medical expenditure (885.25 yuan) in the overall sample. For the medical burden variable, the average burden of 47 people in a “severely ill” condition was 0.925, which is lower than the average of 0.955 in the overall “ill” sample. Clearly, “severely ill” people spend more on medical care, but the reimbursement ratio was higher than average, which is also consistent with the Chinese basic SHI security goals intended to benefit groups with serious illnesses.

### 5.4. Probit Regression Results for Migrant Workers’ Preventive Medical Service Utilization

As shown in Table 5, any insurance can statistically significantly improve the probability of migrant workers’ preventive medical service utilization, especially employment medical care and repeated participation in insurance. As disease severity and education increase, the probability of migrant workers’ utilization of preventive medical services increases, while SRH has no statistically significant effect. Regional differences in preventive medical service utilization also exist for migrant workers; those in the western and central regions attach less importance to such utilization than those in the eastern region.

Qin et al. (2014) illustrate that participating in medical insurance can promote people’s utilization of preventive medical resources by enhancing their health awareness [1]. While public health services and several commercial insurance programs stipulate regular medical examinations, other SHI programs have no clear reimbursement mechanism for routine medical examinations. As a result, young and highly educated people focus more on preventive health care than do elderly people.

## 6. Conclusions and Policy Implications

### 6.1. Main Conclusions

Drawing on data from the RUMiC Project survey, this paper examines the effects of health insurance on migrant workers’ utilization of routine medical services, the medical burden, and the utilization of preventive medical services using a TPM, the Heckman model, the Tobit model, and a probit model. The main conclusions are as follows:

(1) Chinese SHI has not yet covered all migrant workers, and the NRCMS accounts for the main proportion of the types of medical insurance in which they participate. Participating in medical insurance is helpful to increase the probability of migrant workers receiving a medical consultation. Unlike other medical insurance programs that positively affect migrant workers’ medical expenditure, the NRCMS fails to play an effective role. The possible reasons are that the NRCMS requires migrant workers to cover medical expenses and receive reimbursement after returning to their hometowns, which is a cumbersome procedure for migrant workers. SRH and disease severity have significant impacts on the medical expenditure of migrant workers, indicating that their medical care consumption corresponds to maintaining their basic health. In other words, migrant workers usually demand health services after suffering an illness shock, which may exceed their economic means.

(2) Participating in medical insurance significantly reduces migrant workers’ medical burden, especially those with serious illnesses, which is also consistent with the Chinese basic SHI security goals intended to benefit groups with serious illnesses. However, in the overall sample, the proportions of medical subsidies and medical insurance reimbursement are quite low. SHI seems to play a role in decreasing the medical burden, but the coverage and reimbursement proportions are narrow and low in this case. 

(3) Participating in medical insurance (especially employment medical care and repeated participation in insurance) can statistically significantly improve the probability of migrant workers’ preventive medical service utilization. High-income migrant workers have a low probability of illness, but their medical expenses are high once they become ill. On the one hand, a restraining effect may cause migrant workers to fully abandon medical service demands due to constraints on income and SHI. On the other hand, low-income migrant workers are vulnerable in terms of physical health and are consequently prone to illness.

### 6.2. Policy Implications

Based on the findings above, expanding the reimbursement scope of SHI and gradually establishing a comprehensive SHI system to address both serious and minor illnesses seem to be the first priorities for the Chinese government. The basic goal of Chinese SHI is to benefit groups with serious illnesses, which is helpful for migrant workers with these conditions but neglects low-cost outpatient treatment. Migrant workers often self-select into a group with good health conditions such that their main demand for medical services is to address the daily risk of minor illness. Moreover, protecting against both outpatient and inpatient illnesses is more effective than only protecting against serious illnesses since such protection can promote migrant workers’ early consultation and treatment and reduce the phenomenon of ongoing minor illness and burdensome serious illnesses. However, the expansion of the reimbursement scope of SHI corresponds to rising premiums, which is a considerable burden for migrant workers because of their low incomes. Therefore, the government should consider urban and rural economic development and affordability to gradually establish a comprehensive SHI system to protect people with both major and minor illnesses.

Second, reducing the barriers to building an urban‒rural unified SHI system and a convenient and fast reimbursement procedure would be another valuable strategy to address related issues. Doing away with multiple participation in SHI for migrant workers is essential for avoiding overlaps in insurance, preventing medical resource waste, and reducing migrant workers’ economic burden. In addition, establishing a convenient and fast reimbursement procedure for migrant workers is important because of their occupational characteristics, the uncertainty of disease occurrence, and the timeliness of treatment, and will also increase the possibility of migrant workers participating in SHI.

Finally, more than 30% of migrant workers are not covered by any SHI. Among insured migrant workers, the proportion of those who receive medical subsidies is only 11.5%, and the average medical insurance reimbursement ratio is only 5%, indicating that participating in insurance has a significant impact on migrant workers’ medical treatment under the current Chinese SHI system and that the government should not only expand the reimbursement scope of SHI, but also ensure that migrant workers can benefit from SHI, which will improve their opportunities to participate in SHI.

Undeniably, significant differences in the utilization of medical services and medical insurance requirements exist due to the complex composition of migrant worker groups, including differences in age, culture, and income. The uniform standards of SHI will result in some migrant workers abandoning participation in SHI or some SHI participants failing to meet the requirements for effective medical insurance utilization. Therefore, establishing a unified urban‒rural SHI system remains an urgent issue [28,29].

## Figures and Tables

**Table 1:** Sample summary statistics for the selected variables.

Variable	Definition	Overall	Percent (%)	Mean	Std. Dev.
Total medical expenditure	Individual total medical expenditure in the previous year (yuan)	7300		574.08	2897.95
Nonzero medical expenses	Individual total medical expenditure in the previous year above zero (yuan)	4734		885.25	3560.285
Medical burden		4584		0.95	0.164
Preventive medical service utilization	1 = Yes	1865	25.55		
	2 = No	5435	74.45		
Type of medical insurance	0 = No health insurance	2208	30.25		
	1 = NRCMS	4035	55.27		
	2 = employment medical care	337	4.62		
	3 = public health services	162	2.22		
	4 = other single medical insurance	280	3.84		
	5 = multiple insurance	278	3.81		
SRH	Self-rated health (0 = poor, 4 = excellent)	7300		3.10	0.754
Morbidity	1 = Not sick or slightly sick	6389	87.52		
	2 = Fairly sick	605	8.29		
	3 = Seriously sick	306	4.19		
Sex	Dummy variable (1 = male, 0 = female)			0.57	0.495
Age	Range of 16 to 65 years old	7300		32.29	10.293
Marital status (%)	Married	4753	65.11		
	Unmarried	2547	34.89		
Education	0 = Elementary school and below	1158	15.86		
	1 = Middle school	3272	44.82		
	2 = High school	1605	21.99		
	3 = Vocational senior secondary school/specialized secondary school	840	11.51		
	4 = Polytechnic college and above	425	5.82		
Family income	Annual household income (yuan)	7300		2766.40	1685.271
Working hours	Working hours per week on average (hour)	7300		61.15	21.543
Hukou status	0 = Migrant rural household registration	5911	80.97		
	1 = Local rural household registration	1389	19.03		
Region	0 = Eastern China	3788	51.89		
	1 = Central China	2250	30.82		
	2 = Western China	1262	17.29		

**Table 2:** The TPM regression results for migrant workers’ medical expenditure.

Variable	Variable Types	Probability of Migrant Workers’ Consultation	Medical Expenditure
Medical insurance	Insured by NRCMS	0.0987 ***	–0.1321 ***
		(0.0361)	(0.0447)
	Employment medical care	0.1973 **	0.2158 **
		(0.0795)	(0.0890)
	Public health services	0.1581	–0.0784
		(0.1159)	(0.1476)
	Other single medical insurance	0.0940	0.1965 *
		(0.0854)	(0.1053)
	Multiple insurance	0.2516 ***	0.4116 ***
		(0.0904)	(0.1045)
SRH		–0.1817 ***	–0.2776 ***
		(0.0230)	(0.0283)
Morbidity	Fairly sick	1.3565 ***	0.5152 ***
		(0.0937)	(0.0606)
	Seriously sick	2.3421 ***	1.4978 ***
		(0.3412)	(0.0791)
Sex		–0.0586 *	–0.1645 ***
		(0.0328)	(0.0407)
Marital status		0.0230	0.0817
		(0.0446)	(0.0552)
Age		0.0023	0.0095 ***
		(0.0021)	(0.0027)
Education	Junior high school	0.1119 **	0.0174
		(0.0488)	(0.0648)
	High school	0.1555 ***	0.0976
		(0.0550)	(0.0712)
	Technical secondary school	0.2026 ***	0.1138
		(0.0669)	(0.0847)
	Junior college	0.2911 ***	0.0531
		(0.0832)	(0.0993)
Ln (family income)		–0.0596 **	0.0959 ***
		(0.0277)	(0.0360)
Ln (work time)		0.0133	—
		(0.0191)	—
Hukou		–0.0053	—
		(0.0459)	—
Region	Central	0.1646 ***	-0.2854 ***
		(0.0394)	(0.0485)
	Western	0.3989 ***	-0.3785 ***
		(0.0509)	(0.0563)
	Intercept	0.8773 ***	5.2987 ***
		(0.2444)	(0.3066)
*N*		7300	4734
Pseudo *R^2^*/Adj		Pseudo *R^2^* = 0.0916	Adj R-squared = 0.1447
Wald $\chi^{2}$		460.27 ***	—
F		—	50.87

Note: NRCMS represents the New Rural Cooperative Medical Scheme; SRH represents self-rated health. The reference for “medical insurance” is “no medical insurance.” The references for disease severity, sex, marriage, education, Hukou, and region are “not sick or slightly sick,” “female,” “unmarried,” “elementary school and below,” “rural household registration,” and “eastern China,” respectively. *, **, and *** indicate significance at levels of 10%, 5%, and 1%, respectively. The variables in Table 3, Table 4 and Table 5 have the same definitions.

**Table 3:** Heckman selection model of migrant workers’ medical expenditure.

Variable	Variable Types	Medical Expenditure	Decisions for Medical Expenditure
Medical insurance	NRCMS	−0.0205	0.0987 ***
		(0.0749)	(0.0361)
	Employment medical care	0.4268 ***	0.1973 **
		(0.1587)	(0.0796)
	Public health services	0.1116	0.1581
		(0.2037)	(0.1129)
	Other single medical insurance	0.3003 *	0.0940
		(0.1584)	(0.0842)
	Multiple insurance	0.6787 ***	0.2516 ***
		(0.1741)	(0.0891)
SRH		−0.4520 ***	−0.1817 ***
		(0.0694)	(0.0229)
Morbidity	Fairly sick	1.5777 ***	1.3565 ***
		(0.3446)	(0.0929)
	Seriously sick	2.7055 ***	2.3421 ***
		(0.3974)	(0.3302)
Sex		−0.2234 ***	−0.0586 *
		(0.0611)	(0.0328)
Marital status		0.1008	0.0230
		(0.0807)	(0.0451)
Age		0.0118 ***	0.0023
		(0.0037)	(0.0021)
Education	Junior high school	0.1301	0.1119 **
		(0.0952)	(0.0487)
	High school	0.2558 **	0.1555 ***
		(0.1114)	(0.0551)
	Technical secondary school	0.3240 **	0.2026 ***
		(0.1369)	(0.0666)
	Junior college	0.3589 **	0.2911 ***
		(0.1747)	(0.0819)
Ln (family income)		0.0380	−0.0596 **
		(0.0524)	(0.0285)
Ln (work time)		—	0.0133
		—	(0.0194)
Hukou		—	−0.0053
		—	(0.0456)
Region	Central	−0.1012	0.1646 ***
		(0.0903)	(0.0392)
	Western	0.0302	0.3989 ***
		(0.1536)	(0.0508)
	Intercept	4.5144 ***	0.8773 ***
		(0.4806)	(0.2504)
Lambda/Sigma		2.1852 ***	—
		(0.6806)	—
Wald $\chi^{2}$ = 254.27 ***		*N* = 7300	

**Table 4:** Tobit regression results for migrant workers’ medical burden.

Variable	Variable Types	Coefficient	Marginal Effect
Medical insurance	NRCMS	−0.7748 ***	−0.0269 ***
		(0.1080)	(0.0041)
	Employment medical care	−2.0348 ***	−0.0726 ***
		(0.1597)	(0.0067)
	Public health services	−1.5146 ***	−0.0535 ***
		(0.1975)	(0.0078)
	Other single medical insurance	−1.5216 ***	−0.0537 ***
		(0.1729)	(0.0067)
	Multiple insurance	−1.5587 ***	−0.0551 ***
		(0.1584)	(0.0066)
SRH		−0.1358 ***	−0.0048 ***
		(0.0503)	(0.0018)
Morbidity	Fairly sick	−0.2103 *	−0.0074 *
		(0.1137)	(0.0040)
	Seriously sick	−0.5530 ***	−0.0196 ***
		(0.1304)	(0.0048)
Sex		−0.0827	−0.0029
		(0.0753)	(0.0026)
Marital status		0.2176 **	0.0076 **
		(0.1020)	(0.0036)
Age		−0.0121 **	−0.0004 **
		(0.0048)	(0.0002)
Education	Junior high school	−0.0966	−0.0034
		(0.1208)	(0.0042)
	High school	−0.2673 **	−0.0094 **
		(0.1321)	(0.0046)
	Technical secondary school	−0.1913	−0.0067
		(0.1533)	(0.0054)
	Junior college	−0.1375	−0.0048
		(0.1790)	(0.0063)
Ln (family income)		−0.0075	−0.0003
		(0.0618)	(0.0022)
Hukou		−0.1500	−0.0053
		(0.1033)	(0.0036)
Region	Central	0.4814 ***	0.0170 ***
		(0.0955)	(0.0034)
	Western	0.7120 ***	0.0250 ***
		(0.1264)	(0.0045)
	Intercept	4.3401 ***	—
		(0.5596)	—
Lambda/Sigma		1.4800 ***	—
		(0.0645)	—
Observations		4734	
Pseudo *R^2^*		0.0988	
F		17.30	

**Table 5:** Probit regression results for migrant workers’ preventive medical service utilization.

Variable	Variable Types	Coeff./Std. Err.
Medical insurance	NRCMS	0.1730 ***
		(0.0385)
	Employment medical care	0.6025 ***
		(0.0762)
	Public health services	0.3943 ***
		(0.1065)
	Other single medical insurance	0.4164 ***
		(0.0840)
	Multiple insurance	0.6768 ***
		(0.0820)
SRH		0.0177
		(0.0231)
Morbidity	Fairly sick	0.1199 **
		(0.0602)
	Seriously sick	0.2649 ***
		(0.0804)
Sex		0.0173
		(0.0336)
Marital status		−0.0623
		(0.0460)
Age		−0.0152 ***
		(0.0022)
Education	Junior high school	0.0280
		(0.0536)
	High school	0.2029 ***
		(0.0583)
	Technical secondary school	0.2688 ***
		(0.0681)
	Junior college	0.2039 **
		(0.0825)
Ln (family income)		−0.0384
		(0.0307)
Hukou		0.0457
		(0.0480)
Region	Central	−0.2594 ***
		(0.0409)
	Western	−0.4409 ***
		(0.0538)
	Intercept	−0.0790
		(0.2582)
Pseudo *R^2^* = 0.059	Wald $\chi^{2}$ = 462.28 ***	*N* = 7300
